# A Fatal Case of Necrotizing Fasciitis Caused by a Highly Virulent* Escherichia coli* Strain

**DOI:** 10.1155/2016/2796412

**Published:** 2016-05-31

**Authors:** Sadjia Bekal, André Vincent, Alex Lin, Josée Harel, Jean-Charles Côté, Cécile Tremblay

**Affiliations:** ^1^Laboratoire de Santé Publique du Québec, Sainte-Anne-de-Bellevue, QC, Canada H9X 3R5; ^2^Département de Microbiologie, Infectiologie et Immunologie, Université de Montréal, Montréal, QC, Canada H3T 1J4; ^3^Centre Hospitalier Affilié Universitaire, Hôtel-Dieu de Lévis, Lévis, QC, Canada G6V 3Z1; ^4^Faculté de Médecine Vétérinaire, Université de Montréal, Saint-Hyacinthe, QC, Canada J2S 2M2

## Abstract

Necrotizing fasciitis is a serious disease characterized by the necrosis of the subcutaneous tissues and fascia.* E. coli* as the etiologic agent of necrotizing fasciitis is a rare occurrence. A 66-year-old woman underwent total abdominal hysterectomy with bilateral salpingo-oophorectomy. She rapidly developed necrotizing fasciitis which led to her death 68 hours following surgery. An* E. coli* strain was isolated from blood and fascia cultures. DNA microarray revealed the presence of 20 virulence genes.

## 1. Introduction

Necrotizing fasciitis is a severe disease characterized by the necrosis of the subcutaneous tissues and fascia [1]. Despite antibiotherapy and surgical debridement, mortality caused by necrotizing fasciitis can be as high as 30% [1]. It is usually caused by* Streptococcus pyogenes* [2],* Staphylococcus aureus* [3], or a mixture of microorganisms including* Streptococcus*,* S. aureus*, Enterobacteriaceae, and some anaerobes [4–6].* E. coli* is a rare cause of necrotizing fasciitis.

Herein, we report a fatal case of necrotizing fasciitis caused by a highly virulent* E. coli* strain. Its virulence gene complement was determined by DNA microarray.

## 2. Case Presentation

A 66-year-old woman with a prior history of lipectomy and bowel obstruction underwent total abdominal hysterectomy with bilateral salpingo-oophorectomy (TAH-BSO) consecutive to postmenopausal uterine leiomyomata. The complete blood count on admission was within normal range. She received 1 g of cefazolin immediately after surgery and 2 g of cefazolin was added 8 h and 16 h postoperatively. Bruises around the vulva were first noted 18 h following surgery. A bruise on the abdomen and reduced urine flow and oliguria were noted 26 h after surgery despite extensive intravenous rehydration. The patient was afebrile with a blood pressure of 100/80 mmHg and heart rate of 100 beats/min. A new hematological analysis revealed leukocytosis with a white cell count of 13.8 × 10^9^/L (normal: 4.0–10.8 × 10^9^/L). At 48 h after surgery, the patient was fully conscious and alert and felt no unusual pain but her temperature was 38.3°C, blood pressure 80/40 mmHg, and heart rate 130 beats/min. An abdominal ultrasound did not reveal anything specific. Upon examination, grey subcutaneous lesions on both hips and buttocks and on the vulva were noted. She was quickly transferred to the operating room where necrotizing fasciitis covering the abdomen, the thorax, the back, and the groins was confirmed. Surgical debridement was initiated but stopped after three hours given the very large extent of the necrotized tissues. She received clindamycin, ceftriaxone, and immunoglobulins before surgical debridement; fluconazole and vancomycin were added after debridement. The patient died 16 hours later, 68 hours following the TAH-BSO.

## 3. Microbiology and DNA Microarray

Both blood and fascia cultures revealed the presence of a Gram-negative bacillus. It was identified as* E. coli* by API 20E (bioMerieux, Montreal, QC, Canada) with 99.9% certainty and designated LSPQ A134697 (Laboratoire de Santé Publique du Québec, strain A134697). Somatic and flagellar serotypes were identified as O rough:H7, respectively. An antibiotic resistance analysis showed the bacterium to be susceptible to the 14 antibiotics including cefazolin, ceftriaxone, cefotaxime, ceftazidime, cefoxitin, cefotetan, ertapenem, meropenem, imipenem, gentamicin, tobramycin, amikacin, colistin, and ciprofloxacin.

The* E. coli* LSPQ A134697 genome was further analyzed using an oligonucleotide microarray capable of detecting 189* E. coli* virulence genes, according to Bruant et al. [21]. It revealed the presence of at least 20 virulence genes, four encoding toxins,* cnf1* (coding for Cnf1, the cytotoxic necrotizing factor 1),* hlyA* (*α*-hemolysin),* hra* (heat resistant agglutinin), and* vat* (vacuolating autotransporter toxin), six adhesins,* fimA* (subunit of type 1 fimbriae),* fimH* (adhesin),* sfa* (S fimbriae),* papA* (major structural subunit of pilus),* papC* (pilus assembly), and* papG* (specific pilus tip adhesin), one capsule synthesis,* kpsMT*II (group II capsular polysaccharide synthesis), two siderophores,* iroN* (catecholate siderophore) and* fyuA* (*Yersinia* siderophore receptor), one invasin,* ibeA* (virulence factor), and six other activities,* chuA* (heme transport),* iss* (increased serum survival),* ompA* and* ompT* (outer membrane proteins A and T (proteases)),* traT* (surface exclusion), and* yjaA* (stress-response protein). Conversely, other known virulence genes such as* cnf2* (coding for Cnf2, the cytotoxic necrotizing factor 2),* afa* (afimbrial adhesins),* faeG* (F4 fimbrial adhesin),* f17A* (F17 fimbrial subunit),* cdt* (cytolethal distending toxin),* iuc* (aerobactin synthesis),* iutA* (outer membrane receptor protein), and* stx*_*1*_ and* stx*_*2*_ (Shiga toxins I and II) were not detected.

## 4. Discussion

Necrotizing fasciitis caused by* E. coli* is a rare occurrence. Chen et al. [22] showed that* E. coli* was found in 2 out of 126 (1.6%) patients with necrotizing fasciitis caused by a single etiologic agent. Necrotizing fasciitis caused by* E. coli* has been reported from chronically ill patients [23, 24] or infants following surgery [25, 26] but with no mention of the virulence factors present. In a study of 102* E. coli* strains isolated from skin and soft tissue infections, Petkovšek et al. [27] showed that the toxin genes* cnf1* and* hlyA* were present in 32 and 30% of the isolates, respectively, and that only 4% of the strains harbored eight or more virulence factors. Recently, Grimaldi et al. [28] reported an unusual case of necrotizing fasciitis caused by* E. coli*. The isolate was capable of producing the Cnf1 toxin and at least nine other virulence factors. Shaked et al. [29] reported seven cases of* E. coli* necrotizing fasciitis. All seven patients died during hospitalization, three of them during the first 48 h. The* cnf1* gene was found in the* E. coli* strains from two of the latter three cases. It was not researched in the other four cases. No other virulence factors were researched.

We reported here a very rapidly evolving case of an* E. coli*-induced necrotizing fasciitis. It spread through the soft tissues ultimately leading to the patient's death, 50 hours following the first apparition of bruises, despite aggressive antibiotic treatment and the bacterium being sensitive to the antibiotics used. DNA microarray revealed the presence of four toxin genes,* cnf1*, characteristic of necrotoxigenic* E. coli* (NTEC), a pathotype of extraintestinal pathogenic* E. coli* (ExPEC),* hlyA*,* hra*, and* vat*. Six fimbrial adhesins and pili revealed here are cell-surface components that facilitate adherence to other cells, an essential step in pathogenesis in addition to 10 additional virulence genes described in Table 1.

Various subsets of the virulence factors present in* E. coli* LSPQ A134697 have been identified in ExPEC strains, most notably in uropathogenic* E. coli* (UPEC), the causative agent of the vast majority of urinary tract infections, with the exception of the product of the* chuA* gene, present in enterohemorrhagic* E. coli* (EHEC). Together, these virulence factors cover a wide range of activities in toxicity, attachment, invasion, immune suppression, bacterial cell viability, and iron scavenging. The unusually large complement of virulence genes found here identifies this* E. coli* strain as a potentially very aggressive pathogen capable of surviving the host defense mechanisms. Whether some of these virulence factors, alone, or in various combinations with other factors could also provide some novel mechanisms of resistance to the antibiotics used is unknown and worth further investigating.

The current work was the first to report such a large complement of virulence factors in a fatal case of necrotizing fasciitis caused by* E. coli*. It lays the foundations for a better understanding of the various mechanisms involved in the very high pathogenicity of this particular and deadly* E. coli* strain. However, risk factors and interactions with the host can also play a crucial role in infection development and its rapid progression. In this study, the origin of this* E. coli* strain remains unknown. We are now in the process of determining the nucleotide sequence of its genome. It will be compared with other* E. coli* genome sequences, including UPEC and non-UPEC strains isolated from humans and animals.

## Figures and Tables

**Table 1:** List of virulence genes detected in this study and their known function.

Functional category	Gene	Activity/effect
Toxins	*Cnf1*	Causes cell necrosis by activation of GTPases of the Rho family [7] and induces dermal necrosis in rabbits [8]
	*hlyA*	*α*-hemolysin that exhibits pore-forming activities in the membrane of erythrocytes and other cells leading to cell lysis [9]
	*hra*	Promotes agglutination of human erythrocytes and colonic cells, bacterial autoaggregation, enhanced biofilm formation, and aggregative adhesion [10]
	*vat*	Possesses a vacuolating cytotoxic activity on host cells [11]

Adhesins	*fimA*	Mediate a series of signaling events that affect bacterial invasion and promote pro- or anti-inflammatory events [12]
	*fimH*	
	*sfa*	
	*papA*	
	*papC*	
	*papG*	

	*kpsMTII*	Involved in defense avoidance mechanism [13]

Siderophores	*iroN*	Involved in chelation and delivery of iron to bacteria which favor proliferation and enhance pathogenesis [14]
	*fyuA*	

Other activities	*ibeA*	Plays a key role in invasion process, intramacrophage survival, and inflammatory response [15]
	*chuA*	Binds host hemoproteins and transfers the coordinated heme molecule into the bacterium periplasm, where an ABC transport system delivers it to the cytoplasm [16]
	*iss*	Increases resistance to serum [17]
	*ompA*	Serve various functions crucial to cell viability and activity, including structural support, catalysis, active transport, and passive diffusion [18]
	*ompT*	
	*traT*	Confers resistance to the bactericidal activities of serum [19]
	*yjaA*	Involved in the stress response of *E. coli* to hydrogen peroxide and acid as well as in biofilm formation [20]
